# Notes and News

**Published:** 1904-09-03

**Authors:** 


### Notes and News.

The high death-rate in Liverpool is causing some uneasiness locally. No very convincing reason has yet been propounded.

A case of anthrax has occurred in Hampshire, the patient being a man who assisted to kill a bull apparently unsuspected of being diseased at the time, and the hide of which has been disposed of, and is still untraced.

The late Mr. G. McDonald, of Farnham, has bequeathed £1,000 each to the Berkshire Hospital, the Surrey County Hospital, the Aldershot Hospital, the Alton Cottage Hospital, and the Basingstoke Cottage Hospital.

Lord Knutsford gave utterance to some very useful words in favour of the universal teaching of swimming when addressing King Edward's School, Witley, recently. It is astonishing that swimming is the least taught of all athletic exercises. Apart from the fact that a knowledge of swimming tends to the prevention of death from drowning, it is an extremely healthy form of exercise. Every muscle in the body is exercised, whilst the position the body is obliged to assume tends towards the improvement of the figure and bearing.

A dental department is to be opened at the Leeds Dispensary, where the poor will be provided with dentistry at a charge that will cover expenses only. This form of charity is a very useful one. The preservation of the teeth is a great factor in the preservation of health, and yet the poorer classes spend money in this direction with the utmost reluctance. It is quite distressing to see the large percentage of bad teeth, especially amongst the women. As a rule, the poor only resort to the dentist when the pain of continuous toothache is no longer bearable, and when it is cheaper for the sufferer and easier for the dentist to extract the offending tooth.

It has been decided that the employés of the Post Office shall affiliate with the National Committee for the Establishment of Sanatoria for Workers suffering from Tuberculosis, and thus secure provision for members of the Service.

Much regret was expressed at the meeting of the International Congress of Physiologists in Brussels that the honorary president, Sir Michael Foster, was absent for reasons of ill-health. Dr. Heger, the president, read the opening address on Tuesday. The Congress will be held at Turin, Berne, Basle, and Cambridge at future dates.

Sir Joseph Verdin has laid the foundation-stone of an extension of the Victoria Infirmary, Northwich, which is to constitute a Queen Victoria Memorial for Mid-Cheshire. It is estimated to cost £5,000. The Northwich War Committee have subscribed £250 to be added to the building. The whole will provide 12 additional beds, an operating theatre, and new nurses' quarters.

The first International Congress of Education and Home Protection of Infants will be held at Liège in September of next year. The subjects to be discussed are on the face of them mainly educational, but considering the important relations of health to education it is to be hoped that due consideration will be paid by the speakers to the hygienic bearing of their recommendations.

The opening of a block for consumptive paupers at the Union Infirmary, Kettering, is another event which illustrates the awakened sense of responsibility which is making itself apparent amongst boards of guardians. The scheme at Kettering includes a block for chronic cases, a consumption hospital, and an extension of the nurses' home. The buildings designed by Messrs. Gotch and Saunders, have been carried out for a sum between £5,000 and £6,000. Well laid out grounds surround them. The accommodation is for 42 chronic, and 20 acute cases.

The London Hospital has adopted an ingenious contrivance to secure small contributions from the out-patients. A slot machine resembling a clock with a second hand, and bearing the inscription, "Please Keep the London Hospital for a Second," has been placed in the out-patients' department. The introduction of a penny into the slot causes the hand to move forward and record the transaction. Such a device is likely to appeal to the patients, and bring in a considerable revenue to the hospital. Other institutions might usefully follow suit.

A physician, writing to a morning paper, lately gave praise to the rapidly-growing custom of the week-end exodus of working Londoners. He pointed out very truly that the worker who reserves his holiday making until the time for retirement frequently lost the power of enjoying leisure in the meanwhile. But for very many people the week-end holiday is impossible for lack of means. We would suggest that, if the habit becomes more and more fashionable, it may induce a return to a simpler mode of life, which will tend to the welfare of the nation. The luxury which is now regarded as a necessity is one of the evils which is undoubtedly tending towards the degeneration of all classes of English society.

